# MicroRNA regulation in human CD8+ T cell subsets – cytokine exposure alone drives miR-146a expression

**DOI:** 10.1186/s12967-014-0292-0

**Published:** 2014-10-21

**Authors:** Hilary M Sheppard, Daniel Verdon, Anna ES Brooks, Vaughan Feisst, Yu-Yu Joyce Ho, Natalie Lorenz, Vicky Fan, Nigel P Birch, Alicia Didsbury, P Rod Dunbar

**Affiliations:** School of Biological Sciences, University of Auckland, Thomas Building, Auckland, NZ New Zealand; Maurice Wilkins Centre for Molecular Biodiscovery, University of Auckland, Auckland, NZ New Zealand; Bioinformatics Institute, University of Auckland, Auckland, NZ New Zealand; Centre for Brain Research, University of Auckland, Auckland, NZ New Zealand

**Keywords:** microRNAs, T cells, Cytokines, Differentiation, Immunotherapy

## Abstract

**Background:**

microRNAs (miRNAs) are emerging as key regulators of the immune system, but their role in CD8+ T cell differentiation is not well explored. Some evidence suggests that signals from cell surface receptors influence the expression of miRNAs in CD8+ T cells, and may have consequent effects on cell phenotype and function. We set out to investigate whether common gamma chain cytokines modulated human CD8+ T cell expression of miR-146a, which previous studies have associated with different stages of CD8+ differentiation. We also investigated how changes in miR-146a related to other miRNAs that alter with CD8+ differentiation status.

**Methods:**

We treated human CD8+ T cells with the cytokines IL-2, IL-7 or IL-15 either at rest or after stimulation with anti-CD3 and anti-CD28. For some experiments we also purified human CD8+ T cell subsets *ex vivo*. Flow cytometry was used in parallel to assess cell surface memory marker expression. Total RNA from these cells was subjected to microarray analysis and real-time PCR for miRNA expression. Nucleofection studies were performed to assess potential mRNA targets of miR-146a.

**Results:**

We find that miR-146a is up-regulated in naïve CD8+ T cells exposed to IL-2 or IL-15, even in the absence of an activating T cell receptor stimulus, but not when IL-7 is also present. miR-146a expression correlates with a memory phenotype in both *ex vivo* and *in vitro* cultured cells although in our hands overexpression of miR-146a was not sufficient alone to drive a full memory phenotype. In *ex vivo* analysis, miR-146a was one of a small number of miRNAs that was differentially expressed between naïve and memory CD8+ T cells.

**Conclusions:**

miR-146a is emerging as a critical regulator of immune system. Our data shows that miR-146a expression is strongly influenced by the cytokine milieu even in the absence of a T cell receptor stimulus. Our results have implications for studies designed to assess the function of miR-146a, help to define a fingerprint of miRNA expression in CD8+ T cell subsets and may be useful when designing optimal protocols for T cell expansion as efficacy of T cell immunotherapy is correlated with an ‘early’ memory phenotype.

**Electronic supplementary material:**

The online version of this article (doi:10.1186/s12967-014-0292-0) contains supplementary material, which is available to authorized users.

## Background

During an acute intracellular bacterial or viral infection antigen specific CD8+ T cells rapidly proliferate and expand into effector T cells which clear the pathogen. Depending on the strength of stimulating signals received the cells differentiate into specific subsets of memory cells (for a recent review see [1]). The expression of cell surface makers allows these subsets to be identified using polychromatic flow cytometry [2]. Although the exact lineage of the relationship between these subsets remains controversial [3] they can be classified along a progressive path of differentiation based on their phenotype, function, expression of specific transcription factors and more recently their miRNA profile [1,2,4]. However, the molecular circuitry that underlies this differentiation process has only recently begun to be elucidated.

miRNAs are small, 21–23 nucleotide long single-stranded molecules of RNA that function to inhibit gene expression post-transcriptionally [5]. They bind by partial base-pair complementarity primarily to the 3′ UTR of messenger RNAs as part of the RNA induced silencing complex (RISC). It has been predicted that up to 90% of the human transcriptome is regulated by miRNAs [6]. Furthermore it has been proposed that they function to fine tune gene expression [7]. Therefore it is not surprising that the complex system of T cell development which requires careful regulation to achieve immune homeostasis is subject to control by miRNAs, as reviewed by Jeker and Bluestone [4]. An early indication of their involvement in this process was provided by experiments in mice with a conditional deletion of Dicer, a key enzyme in microRNA biogenesis, in the T cell lineage. Impaired peripheral CD8+ T cell development was observed [8]. More recently deletion of Dicer in mature CD8+ T cells in a mouse model suggested a role for miRNAs in the activation, migration and survival of these cells [9].

Roles are also emerging for miRNAs in the differentiation in human CD8+ T cells [10-12]. Such functions for miRNAs have particular relevance to adoptive transfer of T cells for cancer therapy or immune reconstitution following bone marrow and haematopoietic stem cell transplantation. Experiments in mice indicate that the use of less differentiated T cells yields better anti-tumour properties compared to their more differentiated counterparts [13-16]. Clinical grade protocols designed to obtain and maintain these optimum cells are still being defined although it is clear that cytokines used in culture can affect the differentiation status of cells [17]. Typically T cells are cultured in IL-2 which tends to drive cells to a more differentiated phenotype [11,18]. Conversely cytokines such as IL-7 and IL-15 appear to conserve or promote a less differentiated phenotype [19,20]. We set out to understand whether human CD8+ T cells cultured in such cytokines showed changes in specific miRNA molecules that have already been linked to differentiation status. Our hypothesis was that expression of specific miRNAs might vary under different cytokine culture regimes, and that this expression might correlate with – and potentially be responsible for – changes in cell surface phenotype and function. If so, miRNA expression might be informative to the design of protocols used to expand T cells *in vitro* for use in immunotherapy. In addition a memory subset specific miRNA profile could aid identification of the prime T cells for therapeutic use and potentially identify miRNAs that could be used to genetically modify T cells *in vitro* for use in adoptive immunotherapy.

## Methods

### Cell cultures and stimulation

Peripheral blood mononuclear cells (PBMCs) were harvested from healthy donors after informed consent in accordance with methods approved by the local ethics committee (University of Auckland Ethics Committee, NZ). PBMCs were isolated by gradient separation using Lymphoprep™ (Axis-Shield). CD8+ cells were enriched from PBMCs using the CD8+ T Cell Isolation Kit (MiltenyiBiotech) following manufacturer’s instructions. For initial microarray experiments cells were then labelled with anti-CD4-PE, anti-CD45RO-PECy7 and anti-CD28-APC (all from BD Biosciences), anti- CD45RA-PE-TR (Invitrogen), anti-CD8-APC Cy7 (Biolegend) and anti-CCR7 FITC (R&D) fluorescent antibodies. They were then FACS-purified into naïve (CD8+, CCR7+, CD45RA+, CD45RO-), central memory (CD8+, CCR7+, CD45RA-, CD45RO+) and effector memory subsets (CD8+, CCR7-, CD45RA-, CD45RO+) on FACS Aria™ II (BD Biosciences). Post-sorting analysis of purified subsets revealed greater than 98% purity. Cell surface memory phenotyping was performed using the antibodies listed above and CD62L-PerCpCy5.5 (Biolegend).

For subsequent validation experiments CD3+ or CD8+ cells were enriched from PBMCs using the Pan T Cell Isolation Kit II or CD8 Isolation Kit respectively (MiltenyiBiotech) following manufacturer’s instructions. To further enrich for the naïve cell fraction (CD45RO-) CD45RO microbeads were used (MiltenyiBiotech). For expansion experiments T cells were activated with anti-CD3/CD28-conjugated magnetic beads (Expander Beads, Invitrogen) in 1:1 bead/T-cell ratio in RPMI medium supplemented with 5% human serum and IL-2 and IL-12 at 10 ng/ml. After 48 hours beads were removed and cells were grown in either IL-2 at 10 ng/ml, IL-7 at 10 ng/ml or IL-15 at 10 ng/ml or concentrations as stated in the text. For culture in cytokine in the absence of a TCR stimulus naïve cells were rested in RS5 with either IL-2 or IL-15 +/− IL-7 or IL-7 alone. All cytokines were supplied by Peprotech. Cytokines and medium were replaced every 3–4 days.

### Microarrays

FACS sorted cells were washed once in ice-cold PBS and total RNA was purified using the miRVANA kit (Ambion). RNA integrity was assessed using a bioanalyser (Agilent). 500 ngs of RNA were reverse transcribed and labelled using the Flash Tag Biotin HSR kit (Genisphere) and hybridised to Gene Chip miRNA Arrays 1.0 (Affymetrix) according to manufacturer’s protocols. Fluorescent signals were recorded by an Affymetrix scanner 3000 using Gene Chip Operating Software. The statistical software program R was used to analyse the results. The data was pre-processed and normalised using RMA from the R package [21]. The R package limma was used to check for differential expression, and an empirical Bayes method was used to moderate the t-statistic. In order to adjust for multiple testing, the Benjamini-Hochberg method was used to correct the p-values, and adjusted p-values are reported in the text. The human subset of the results was extracted and all subsequent plots were made on the human transcripts. Microarray data has been deposited in the Gene Expression Omnibus (GEO) database and can be accessed via accession no. GSE54867.

### qRT-PCR

Total RNA was purified using the miRVANA kit (Ambion) or where stated with RNA-GEM Tissue Plus (ZyGem). To analyse the expression of specific miRNAs individual cDNAs were prepared from 10 ngs miRVANA RNA using the Taqman miRNA RT kit (ABI) and specific Taqman small RNA primers (ABI) according to manufacturer’s instruction. Real-time PCR reactions were prepared with Taqman FAST mastermix and run on an ABI Prism HT 7900 machine (Applied Biosystems). RNU44 was used as an internal control and relative expression was calculated as ∆∆Ct. To analyse the expression of specific mRNAs total cDNA was prepared using the Superscript first strand synthesis cDNA kit (Invitrogen). Real-time PCR reactions were prepared using 10 ngs total cDNA with Taqman probes (ABI) and run as above. HPRT and B2M were used as internal control genes and relative expression was calculated as ∆∆Ct. RNA-GEM Tissue Plus was used according to manufacturer’s instructions. 500,000 cells were lysed in 50 uls lysis solution. 2uls of the resulting lysate was used directly in cDNA synthesis reactions for miRNA and total cDNA.

### Nucleofection

Cells were electroporated using the Amaxa Human T Cell Nucleofector® Kit (Lonza) according to manufacturer’s instructions for unstimulated human T cells. Per transfection, 5 million cells were combined with 300nM siRNA or miRNA mimic (miRVANA miRNA mimic, Ambion) and pulsed in a Nucleofector II device using program V-024. Equivalent amounts of fluorescently labelled Block iT siRNA (Invitrogen) was used to assess electroporation efficiency in separate control samples. After 48 hours total RNA was purified using the miRVANA kit (Ambion).

### Analysis of CD45 isoforms by PCR

Total RNA was purified using the miRVANA kit (Ambion) and CD45 isoform expression was assessed by PCR as described by ten Dam *et al.* [22]. In brief, first strand cDNA was prepared using primer LCA9 (5′-GTAATCCACAGTGATGTTTGC-3′) with 250 ng total RNA and the Superscript first strand synthesis cDNA kit (Invitrogen). cDNA was then amplified using primers LCA2 (5′-ATTGGATCCGCTGACTTCCAGATATGACC-3′) and LCA7 (5′-CCGAGATCTTCAGAGGCATTAAGGTAGGC-3′). PCR products were visualised on a 2% agarose gel.

## Results

### Differential memory phenotype and miR-146a expression in CD8+ T cells expanded in different cytokines

**Figure 1:**
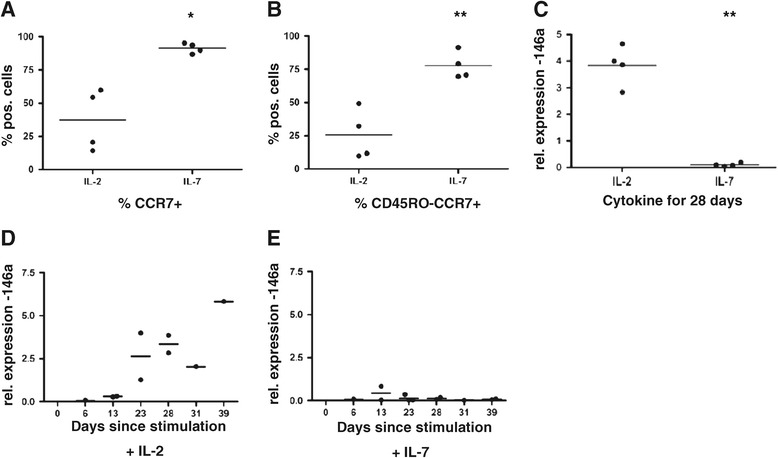
**miR-146a expression correlates with a memory phenotype and exposure to IL-2 in naïve T cells.** Naïve CD8+ T cells were stimulated with anti-CD3/28 magnetic beads and cultured in the presence of either 10 ng/ml IL-2 or IL-7. At 28 days post stimulation cells were harvested and either subjected to FACS analysis to assess expression levels of T cell memory markers CD45RO and CCR7 or assessed for miR-146a expression levels by RT-PCR. **(A)** Graph showing % positive cells for CCR7+ and **(B)** CCR7 + CD45RO- as assessed by FACS analysis. Each data point represents one donor, n = 4. Statistical significance was analysed using GraphPad Prism software using the Student’s *t*-test. **(C)** RT-PCR relative expression data for miR-146a at day 28 post stimulation for the cells phenotyped in panels A and B. Each data point represents the average of triplicates from an individual donor, n = 4. Statistical significance was analysed using GraphPad Prism software on non-normalised expression data using the Student’s *t*-test. Results with a p value <0.05 were considered to be statistically significant. * = p <0.05; ** = p <0.01. **(D)** Naïve T cells were stimulated as in panel C in the presence of 10 ng/ml IL-2. At various time points post-stimulation cells were harvested and miR-146a expression levels were assessed by RT-PCR. **(E)** As **(D)** but cells were grown in the presence of 10 ng/ml IL-7. Each data point represents the average of triplicates from an individual donor. The data in panels D and E represent collated data from 4 donors (5 donors for IL-7) each tested at between 1 – 4 time points.

To investigate the effects of different cytokines on T cell phenotype and miRNA expression, we first developed a system in which T cells could be cultured with or without exogenous IL-2. We took naïve CD8+ T cells and stimulated them with CD3/28 beads and expanded them either in the presence of IL-7 or IL-2 alone for 28 days. We then assessed the cell surface phenotype of our *in vitro* stimulated cells to establish if culture in IL-2 versus IL-7 caused a modulation of receptors associated with T cell survival and effector function. Human CD8+ T cells can be assigned into at least four subsets based on their expression of specific cell-surface markers which correlate with their differentiation status. These subsets and markers include naïve (N = CCR7+, CD45RA+, CD45RO-, CD28+, CD62L+), central memory (Tcm = CCR7+, CD45RA-, CD45RO+, CD28+, CD62L+), effector memory (Tem = CCR7-, CD45RA-, CD45RO+, CD28+, CD62L-) and effector memory CD45RA + cells (TemRA = CCR7-, CD45RA+, CD45RO-, CD28-, CD62L-). As is shown in Figure 1A-B we observed a significantly lower proportion of CCR7+ and CCR7 + CD45RO- cells (*i.e.* indicative of a naïve-like phenotype) being retained following CD3/CD28 bead stimulation and culture for 28 days in IL-2 versus IL-7.

In order to assess miRNA expression we prepared RNA from cells exposed to IL-2 and IL-7 for 28 days as above and subjected it to microarray analysis using GeneChip miRNA Arrays 1.0 (Affymetrix). The arrays contain 46,228 probe sets representing over 6703 miRNAs. We confined our analysis to the 847 human miRNAs represented on the chip. A normalised log2 fluorescence value of 4.0 was set as our arbitrary cut off for expression *i.e.* a value <4.0 was counted as ‘not expressed’. Although expression of most of the miRNAs tested were not affected by the different culture conditions, miR-146a showed an average fold difference in expression of >124 (data not shown). This result was validated using RT-PCR (using the same cells phenotyped in Figure 1A-B) and the positive relationship between miR-146a expression and IL-2 exposure is shown in Figure 1C. A time-course was performed to establish at what point miR-146a expression was induced. The differential expression in IL-2 vs IL-7 became apparent between 13–23 days post-stimulation and tended to increase in the presence of IL-2 over time (Figure 1D and E).

### miR-146a expression is up-regulated in the presence of IL-2 but not IL-7, even in the absence of a TCR stimulus

**Figure 2:**
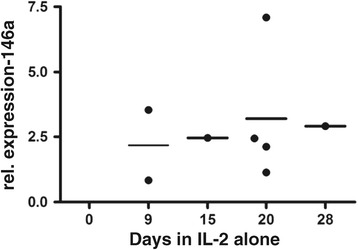
**miR-146a is up-regulated by IL-2 exposure alone without an activating TCR stimulation.** Naïve cells were not stimulated, but following purification were cultured in the presence of 10 ng/ml IL-2. At various time points cells were harvested and miR-146a expression levels were assessed by RT-PCR. Each data point represents the average of triplicates from an individual donor. The data represents collated data from 4 donors each tested at between 1 – 4 time points.

As described above, miR-146a was up-regulated in human CD8+ cells stimulated and cultured in the presence of IL-2, consistent with previous report in human CD4+ cells [23,24], Jurkat cells [24] and mouse CD8+ cells [23,25]. However it was not clear whether the up-regulation required a TCR signal, or was dependent upon exposure to IL-2, or both. We therefore next asked if IL-2 alone was sufficient to drive the expression of this miRNA. Naïve cells were isolated and rested for up to 28 days in IL-2. As shown in Figure 2 miR-146a was up-regulated by IL-2 alone, in the absence of an exogenous TCR stimulus. Levels of expression were in a similar range to those observed in the presence of IL-2 with a TCR stimulus (cf. Figures 2 to 1C and D).

### miR-146a expression is up-regulated by IL-2 or IL-15 but inhibited if IL-7 is also present

**Figure 3:**
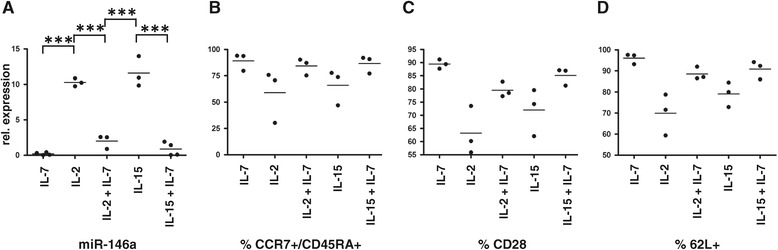
**miR-146a expression is up-regulated by IL-2 or IL-15 but inhibited if IL-7 is also present. (A)** Naïve T cells were cultured in the presence of IL-7, IL-2, IL-7 and IL-2, IL-15 or IL-7 and IL-15, all at 10 ng/ml. 28 days later cells were harvested and assessed for miR-146a expression levels by RT-PCR. Each data point represents the average of triplicates from an individual donor, n = 3 or 4. Statistical significance was analysed using GraphPad Prism software using one way ANOVA test (on paired data) with Tukey’s multiple comparison correction applied. Results with a p value <0.05 were considered to be statistically significant. *** = p <0.001. **(B - D)** Graphs showing % positive cells for CCR7+/CD45-RA+, CD28+ or CD62L + as assessed by FACS analysis. Each data point represents data from one donor, n = 3.

The IL-2 receptor is made up of 3 subunits: alpha (CD25), beta (CD122) and gamma (CD132). The gamma chain is common to the receptors of 6 cytokines including IL-2, IL-15 and IL-7, the beta chain is shared only by IL-2 and IL-15 whilst the alpha chain is unique to IL-2 alone. Therefore to assess the involvement of signalling via the beta chain in the absence of a TCR stimulus we next asked if IL-15 could also induce expression of miR-146a in the same way as IL-2. As is shown in Figure 3A naïve cells exposed to IL-15 for 28 days express high levels of miR-146a comparable to cells exposed to IL-2 alone, and in both cases significantly higher levels than in cells exposed to IL-7 alone, where expression is negligible. Interestingly when cells are cultured in IL-2 or IL-15 plus IL-7 there is no significant up-regulation of miR-146a, suggesting that the presence of IL-7 inhibits expression of this miRNA. This finding is consistent with the data in Figure 1, where the major difference between the two cultures is exposure to IL-7. Concomitant with an up-regulation of miR-146a we also observed movement towards a cell-surface memory phenotype with a decrease in the percentage of CCR7 + CD45RA+, CD28+ and CD62L + cells (Figure 3B-D).

### Antigen exposure *in vivo* strongly upregulates miRNA-146a in human CD8+ T cells

If IL-2 exposure upregulates miRNA-146a *in vivo* differences might be expected between naïve and memory T cell subsets in their expression of miRNA-146a. However this might be modulated by exposure to homeostatic IL-7 and IL-15, which could theoretically act to respectively suppress or enhance miR-146a in both naïve and memory CD8+ T cells. Previous studies have indicated that miR-146a levels are upregulated in CD8+ T cell memory subsets [10,24]. To confirm this, and to investigate the strength of miR-146a modulation in CD8+ T cell subsets *in vivo* relative to other microRNAs, we carried out micro-array analysis on sorted CD8+ T cell subsets. Human CD8+ T cells were sorted *ex vivo* to ≥98% purity from 3 healthy donors into the following subsets: naïve (N = CCR7+, CD45RA+, CD45RO-), central memory (Tcm = CCR7+, CD45RA-, CD45RO+), effector memory (Tem = CCR7-, CD45RA-, CD45RO+) and effector memory CD45RA + (TemRA = CCR7-, CD45RA+, CD45RO-). The 3 donors were selected from a wider pool of donors to select for those with high proportions of N and Tcm cells as we wanted to examine whether the earliest events of antigen exposure were capable of modulating miRNA-146a expression in early memory cells compared to naïve cells.

We analysed the expression of miRNAs in N, Tcm and Tem CD8+ T cell subsets using GeneChip miRNA Arrays 1.0 (Affymetrix). We first asked how many miRNAs are expressed overall in these subsets of CD8+ T cells. 85 miRNAs were detected in all 3 subsets, using a normalised log2 fluorescence value of ≥ 4.0 across all 3 donors as our threshold for expression (see Additional file 1: Table S1). Using the same criteria to detect miRNAs expressed in any subset, 112 miRNAs were expressed (see Additional file 2: Table S2). Our analysis therefore confirms that a limited subset of the miRNAs tested (13.2%) are expressed in human CD8+ T cells and that there is differential expression of specific miRNAs between CD8+ T cell subsets. We found 29 miRNAs that are differentially expressed between T cell subsets with statistical significance (p value <0.05, see Table 1). Unsupervised clustering (data not shown) suggests that naïve CD8+ T cells from different individual donors are more similar to each other than to memory T cell subsets from the same donor. Tcm and Tem do not cluster as discrete subsets which suggest that there is more of a continuum between these subsets. Interestingly there were no statistically significant differences observed between Tcm and Tem subsets. This also reflects high inter-donor variability.

**Table 1:** **P values for miRNAs significantly differentially expressed when comparing naïve to Tcm or Tem subsets**

**miRNAs up-regulated with differentiation**			
	**Tcm-naive**	**Tem-naive**	**Tem-Tcm**
hsa-miR-146a	**0.010**	0.075	0.447
hsa-miR-221	**0.023**	0.130	0.737
hsa-miR-222	**0.023**	0.056	0.912
hsa-miR-23a	**0.030**	**0.008**	0.773
hsa-miR-24	**0.046**	**0.005**	0.413
hsa-miR-15a	0.742	**0.041**	0.457
hsa-miR-22	0.521	**0.043**	0.532
hsa-miR-28-5p	0.668	**0.044**	0.518
hsa-miR-663	0.340	**0.034**	0.600
**miRNAs down-regulated with differentiation**			
	**Tcm-naive**	**Tem-naive**	**Tem-Tcm**
hsa-miR-181a	**0.006**	**0.006**	0.800
hsa-miR-874	**0.013**	**0.006**	0.929
hsa-miR-92a	**0.014**	**0.013**	0.983
hsa-miR-378	**0.015**	**0.015**	0.973
hsa-miR-151-5p	**0.015**	**0.005**	0.651
hsa-miR-502-3p	**0.015**	0.152	0.518
hsa-miR-181b	**0.021**	**0.040**	0.936
hsa-miR-320d	**0.022**	**0.009**	0.912
hsa-miR-181c	**0.022**	**0.034**	0.976
hsa-miR-146b-3p	**0.023**	**0.012**	0.951
hsa-miR-342-3p	**0.027**	0.059	0.929
hsa-miR-1271	**0.028**	0.619	0.457
hsa-miR-146b-5p	**0.032**	0.093	0.902
hsa-miR-324-3p	**0.038**	0.362	0.651
hsa-miR-1275	**0.040**	0.609	0.518
hsa-miR-194	**0.044**	0.072	0.975
hsa-miR-342-5p	**0.048**	0.146	0.903
hsa-miR-20b	0.052	**0.038**	0.981
hsa-miR-192	0.157	**0.034**	0.814
hsa-miR-31	0.950	**0.009**	0.167

p values are shown for miRNAs that were statistically significantly up or down regulated when comparing naïve cells to either the Tcm or Tem subsets using Affymetrix miRNA microarrays.

Figures in bold represent p values <0.05.

**Figure 4:**
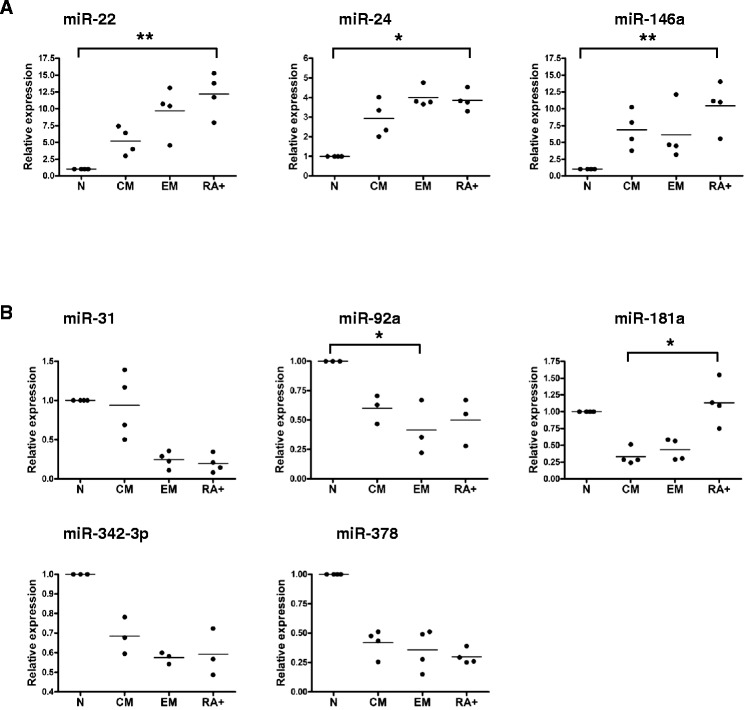
**Specific miRNAs are differentially expressed in CD8+ T cell subsets.** The expression levels of nine miRNAs identified by microarray analysis as being significantly differentially expressed between sorted naïve (N), central memory (CM) and effector memory (EM) T cell subsets from 3 healthy humans donors were validated in a further 3 or 4 new donors by RT-PCR. Panel **(A)** shows miRNAs which were up regulated and **(B)** shows miRNAs which were down regulated when comparing naïve to Tcm cells in microarray experiments. Each data point represents the average of triplicates from an individual donor and is expressed relative to levels in naïve cells which are set to 1. Statistical significance was analysed using GraphPad Prism software on non-normalised expression data using the Friedman test (on paired, non-parametric data) with Dunn’s multiple comparison correction applied. Results with a p value <0.05 were considered to be statistically significant. * = p <0.05; ** = p <0.01.

Given the similar profiles in the naïve CD8+ T cells of all 3 donors, we next asked which miRNAs showed the largest differences between naïve cells and either of the memory subsets *i.e.* which had been most strongly and consistently modulated by a history of antigen exposure. There were 9 statistically significant up-regulated miRNAs when comparing naïve cells to one or both of the memory subsets with the most significant hit being miR-146a (when comparing naïve to Tcm subsets - see Table 1). Up-regulation was confirmed by RT-PCR in four new donors for each of the miRNAs that we validated in this way (namely miR-22, −24 and -146a) (see Figure 4A - TemRA cells were also included in this analysis). The trend in increased expression with differentiation extended to the TemRA subset in which miRNA expression was significantly higher than in the naïve subset in each case. However, due to high donor variability statistical significance was not achieved when comparing naïve cells to the Tcm or Tem subsets. Notably the RT-PCR results indicate that expression levels of miR-146a were 4x and 50 x higher overall than the expression levels of miR-24 and miR-22 respectively (data not shown).

20 miRNAs were statistically significantly down-regulated when comparing N cells to one or both of the memory subsets (see Table 1). As observed with the up-regulated miRNAs there tended to be a trend toward down-regulation of these miRNAs across both memory subsets. Again down-regulation was confirmed by RT-PCR in four new donors for each of the miRNAs we validated in this way (miR-31, −92a, −181, −342-3p and −378, see Figure 4B). Due to strong inter-donor variability statistical significance was only achieved in 2 out of the 5 miRNAs tested. However, in each case there was a definite downward trend in expression over the memory T cell subsets compared to the naïve subset which tended to extend to the TemRA subset also. miR-31 exhibited a slightly different expression pattern with levels in the Tcm subset being more similar to the naïve subset than to the Tem subset (in both microarray and RT-PCR experiments). miR-181 also showed a unique profile with significant up-regulation of expression in TemRA cells compared to Tcm cells in all 4 donors tested. Collectively these data show that a limited number of miRNAs including miR-146a are differentially expressed between T cell subsets and are likely to be involved in their differentiation status.

**Figure 5:**
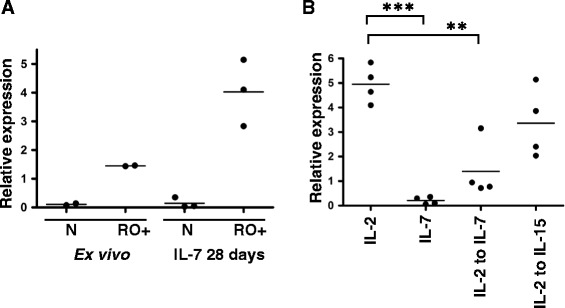
**miR-146a expression is modulated by cytokine exposure in naïve cells but constitutive in memory cells. (A)** The relative expression of miR-146a was assessed by RT-PCR in freshly purified uncultured *ex vivo* memory cells or memory cells that had been cultured for 28 days in the presence of 10 ng/ml IL-7. Each data point represents the average of triplicates from an individual donor, n = 2 or 3. **(B)** Naïve T cells were cultured in the presence of IL-2 or IL-7 for 14 days and then either maintained in the same cytokine or transferred into IL-7 or IL-15 for a further 7 days, all at 10 ng/ml. Cells were harvested and assessed for miR-146a expression levels by RT-PCR. Each data point represents the average of triplicates from an individual donor, n = 4. Statistical significance was analysed using GraphPad Prism software using one way ANOVA test with Bonferroni’s multiple comparison correction applied. Results with a p value <0.05 were considered to be statistically significant. ** = p <0.01, *** = p <0.001.

In order to assess the effect of IL-7 on miR-146a expression in *ex vivo* memory cells, CD45-RO+ cells were cultured in the presence of IL-7 for 28 days. In this situation miR-146a expression was retained, if not enhanced, compared to the expression levels detected in uncultured *ex vivo* memory cells (Figure 5A). We therefore examined whether miRNA-146a expression became constitutive following exposure of naive cells to IL-2, or whether continued signalling through CD122 was required to maintain miRNA-146a expression in these cells. Naïve cells exposed to IL-2 for 14 days were transferred into either IL-7 or IL-15 for a further 7 days and miR-146a expression was assessed (Figure 5B). There was a statistically significant drop in miR-146a expression when cells were moved from IL-2 and into IL-7. However, if the cells are moved into IL-15 expression levels remain high. These data suggest that in these naïve cells the continued presence of IL-2 or IL-15 is required to maintain the high levels of miR-146a expression. Conversely *ex vivo* CD8+ memory cells cultured for 21 days in IL-7 alone retain high levels of miR-146a expression (Figure 5A). This suggests that at some point in the differentiation process miR-146a expression becomes constitutively expressed.

### CCR7, FADD and TRAF6 are targets of miR-146a

**Figure 6:**
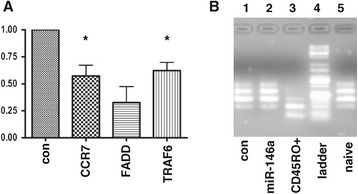
**CCR7, FADD and TRAF6 are targets of miR-146a.** Naïve CD8+ or CD8+/CD4+ T cells were nucleofected with either miR-146a mimic or scrambled control. After 48 hours culture in 10 ng/ml IL-7 cells were harvested and either **(A)** subjected to RT-PCR analysis to assess expression of putative targets of miR-146a namely CCR7, FADD and TRAF6 or **(B)** PCR was performed to analyse for the presence of various CD45 isoforms in cells following nucleofection. **(A)** Each bar represents the average of triplicates from three separate experiments and is expressed relative to the expression levels for each gene in cells nucleofected with the scrambled control (con) which is set to 1. Error bars represent standard deviation. A paired student’s *t* test was used to assess significance on non-normalised data * = p <0.05; ** = p <0.01. **(B)** Following CD45 specific cDNA synthesis and PCR, samples were visualised on an agarose gel. Lane 1 = scrambled control, lane 2 = miR-146a mimic. As a comparison CD45 isoforms present in either CD45RO + memory cells or *ex vivo* naïve were also assessed (lanes 3 and 5 respectively). Lane 4 = DNA ladder.

As miR-146a expression correlated with a memory phenotype in both *ex vivo* and *in vitro* cultured cells we next asked if miR-146a expression alone is sufficient to drive a memory phenotype. To answer this we nucleofected naïve T cells with a miR-146a mimic and assessed the expression of cell surface memory markers (CD45RA, CD45RO and CCR7) by FACS. Cellular uptake of fluorescently-labelled control reagents suggested high uptake (>95%) of the mimic at 48 hours post-nucleofection (data not shown). However, using flow cytometry we observed no detectable effect on cell surface memory markers at this time point (data not shown). This was possibly due to the time of sampling as an analysis of CCR7 mRNA expression did suggest a significant down regulation in expression at 48 hours (see Figure 6A). In addition TRAF6 expression, a previously validated target for miR-146a [23,25], was significantly down regulated at 48 hours post-nucleofection (Figure 6A). We also observed down-regulation in the expression levels of FADD mRNA, another predicted target of miR-146a [24]. We assessed CD45 isoform expression at the mRNA level (Figure 6B). However, CD45 isoform expression was not affected following nucleofection with miR-146a mimic and remained similar to those isoforms detected in *ex vivo* naïve cells rather than those present in purified *ex vivo* memory cells. These results suggest that over-expression of miR-146a alone may not be sufficient to drive a cell towards a memory phenotype. Alternatively it could be that prolonged expression by miR-146a is required which could not be achieved using this experimental system.

## Discussion

We find that miR-146a is highly expressed in cells cultured *in vitro* in the presence of IL-2 or IL-15, even in the absence of a TCR stimulus. In addition, expression of this microRNA correlates with a cell surface memory phenotype *in vitro* and in *ex vivo* cells. Interestingly, miR-146a expression is suppressed in the presence of IL-7. Our results therefore show that miRNA expression can be significantly affected by the presence of cytokines alone. This has implications when designing expansion protocols for T cells for use in immunotherapy.

There have been some recent reports indicating that the expression of other microRNAs appears to be influenced by cytokines [11,26-28]. However, miR-182 in mouse CD4+ T cells for example, requires both a TCR and IL-2 to be induced; IL-2 alone was insufficient [26]. Expression of miR-182 drops 4 days post stimulation. In concordance with that result we did not observe up-regulation of miR-182 in our array experiments on stimulated cells exposed to IL-2 for 28 days. Rapid induction of miR-146a has been shown in human monocytes in response to LPS and various microbial components (expression was induced within hours and assessed up to 14 hours post stimulation) [29]. miR-146a has also been shown to be rapidly induced in response to IL-1β in human lung alveolar epithelial cells (expression was induced within hours and assessed up to 24 hours post stimulation) [30]. However, to our knowledge this paper represents the first report to show significant modulation of expression of a microRNA in T cells by prolonged exposure to cytokine alone. miR-146a has also been reported to be induced post a TCR stimulus [23-25] in T cells but we did not observe this in our experiments (data not shown). However we note that our data agrees with data reported for primary human CD4+ cells [24] where up-regulation of miR-146a was only observed at between 12–14 days post TCR stimulation. Our data suggest that TCR signalling is not causative for miR-146a expression and that it is possibly the IL-2 produced by T cells following TCR stimulation that up-regulates miR-146a.

miR-146a is emerging as a critical regulator of both the innate and adaptive immune systems [31]. Its function in T cells appears to be to regulate TCR-driven NFkB activation by targeting the signal transducers TRAF6 and IRAK1, such that T cells in mice lacking miR-146a are hyperactive [25]. More recently it has been implicated in T cell differentiation [23] which is in concordance with our data. Although nucleofection utilising a miR-146a mimic and human naïve CD8+ cells was not sufficient to drive a full memory phenotype we did observe down-regulation of CCR7 and TRAF6 mRNA comparable to other recent observations [23]. As CCR7 is not predicted to be a direct target of miR-146a this is likely to be an indirect effect. In addition we note that *ex vivo* Tcm which are CCR7+ and Tem which are CCR7- appear to express similar levels of miR-146a. Nevertheless the reductionist approach of nucleofection suggests that one component of CCR7 modulation is miR-146a expression. TRAF6 has been implicated in the generation of memory cells in mice [32]. As TRAF6 has been shown to be a direct target for miR-146a [23,25] this supports the idea that miR-146a is involved in T cell memory formation or function. However, further experimentation with sustained overexpression of miR-146a will be required to more fully examine the role of this microRNA in T cell memory.

Our nucleofection results also validated FADD as another potential direct target for miR-146a in line with other recent observations [24]. FADD is an adaptor protein involved in FAS-mediated apoptosis pathway. However, we found no correlation between miR-146a expression and a reduction in apoptosis in our naïve T cells cultured in IL-2 (data not shown), which is in concordance with the findings of others [23].

Our results suggest that both IL-15 and IL-2 can drive miR-146a expression whilst IL-7 does not. IL-2, IL-7 and IL-15 share the CD132 subunit in their receptors but only IL-2 and IL-15 share the CD122 receptor subunit. This suggests that signalling via CD122, the shared receptor chain common to both of these cytokines, may be driving expression of miR-146a. Concomitant with increased expression of miR-146a we also observed a move towards a cell-surface memory phenotype even in the absence of a TCR stimulus. The effect is less marked than in the presence of a TCR and we hypothesise that both miR-146a expression and cell division are required to achieve a memory phenotype. It is interesting to note that signalling via CD122 has been implicated in T cell memory development [33] and further supports the hypothesis that miR-146a is involved in formation of a memory phenotype. The fact that IL-7 appears to inhibit the IL-2 or IL-15 driven upregulation of miR-146a indicates that there may be competition for the CD132 subunit shared by all three cytokines, as has been suggested previously [34]. We note that miR-146a expression is somewhat delayed in cells exposed to IL-2 in the presence of a TCR stimulus compared to its absence (compare Figure 1E with Figure 2). CD25 is the alpha chain of the IL-2 receptor, expression of which has been shown to be up-regulated for a number of days following a TCR stimulus [35]. CD25 up-regulation may interfere with the CD122 driven signalling which is most likely driving expression of miR-146a. This up-regulation would not occur in cells exposed to IL-2 in the absence of a TCR stimulus, and this may explain the more rapid expression of miRNA-146a in these cells.

A new T cell subset, stem cell-like memory T cells (Tscm), has recently been identified [13]. It was reported that naïve T cells expanded in the presence of IL-15 and IL-7 retain an early stem cell memory phenotype *i.e.* CD45RA + CD45R0 + CD62L + CCR7 + CD95+ [19]. The presence of IL-7 was found to be unique in its ability to instruct the T cells to a Tscm phenotype, while IL-15 and CD28 co-stimulation appeared to be critical to optimum expansion of the cells. Similar to our observations it was reported that expansion in the presence of IL-2 or IL-15 drive a more memory-like phenotype [19]. In combination with our results we propose that the presence of IL-7 is required to inhibit the expression of miR-146a and therefore promote an early stem cell phenotype. Therefore expression of miR-146a may be a critical molecular modulator underlying the generation of the optimum T cell for use in immunotherapy.

Our microarray studies on *ex vivo* CD8+ T cell memory subsets confirms a correlation between miR-146a expression and a cell surface memory phenotype as has been observed by others [23-25]. The array data also showed that there is differential expression of 112 miRNAs in the three CD8+ T cell subsets we examined. As each miRNA can affect multiple target genes and a single gene can be targeted by multiple miRNAs [36] this is likely to affect subsequent gene expression and affect subset function. The number of miRNAs we identified is similar to that observed in another study using a RT-PCR array approach [10]. Our data expands this earlier data set and helps to define the miRNA fingerprint present in human CD8+ memory subtypes. Up or down-regulation of individual miRNA expression was confirmed in each case where RT-PCR was used to validate microarray results. Although results were not always statistically significant this reflects high levels of inter-donor variability as has been observed previously [10]. In general the trend of expression was consistent across the 4 subsets tested *i.e.* increasing or decreasing progressively from naive through to Tem subsets.

## Conclusion

In conclusion we show that expression of miR-146a and cell surface memory phenotype is influenced by the cytokine environment *in vitro*, independent of an activating TCR. Our array data helps to refine a miRNA expression fingerprint that defines specific human memory CD8+ T cell subsets to which *in vitro* cultured cells can be compared. These results have implications to studies designed to assess the function of miR-146a. In addition they are informative to the design of protocols used to expand CD8+ T cells for immunotherapy.

## Additional files

Additional file 1:
**miRNAs expressed in CD8+ T cells.** The average expression of miRNAs across all CD8+ T cell subsets from 3 donors is shown with a normalised log2 fluorescence value > 4.0. Data is derived from Affymetrix microarray experiments. Additional file 2:
**miRNAs expressed in CD8+ T cell subsets.** The average expression of miRNAs in individual CD8+ T cell subsets from 3 donors is shown with a normalised log2 fluorescence value > 4.0. Data is derived from Affymetrix microarray experiments.

## Abbreviations

IL: Interleukin
N: Naïve T cell
PBMC: Peripheral blood mononuclear cells
miRNA: microRNA
RISC: RNA induced silencing complex
Tcm: Central memory T cell
TCR: T cell receptor
Tem: Effector memory T cell
TemRA: Effector memory CD45RA + T cell
Tscm: Stem-cell like memory T cell
